# Primary renal Ewing’s sarcoma in a young male treated completely by surgical excision without chemotherapeutic agents: Case report

**DOI:** 10.1016/j.ijscr.2019.09.016

**Published:** 2019-09-23

**Authors:** Khalid M. Abdalla, Nour A. Tashtush, Abdelwahab J. Aleshawi, Ghassan Talahmeh, Khaled Z. Alawneh

**Affiliations:** a Department of Diagnostic and Interventional Radiology, Faculty of Medicine, Jordan University of Science and Technology, Irbid, 22110, Jordan; b Faculty of Medicine, Jordan University of Science and Technology, Irbid, 22110, Jordan; c Department of General Surgery and Urology, Prince Hamza Hospital, Ministry of Health, Amman, 11947, Jordan

**Keywords:** Nephrectomy, CD99, Ewing, Round cell tumor

## Abstract

**Introduction:**

Primary renal Ewing’s sarcoma is a rare tumor with dismal prognosis. The main treatment modality of treatment is surgical excision with adjuvant or neoadjuvant chemotherapy.

**Case presentation:**

We present a case of primary renal EWS in 27-year-old male. He presented to our center complaining of three days history of severe left flank pain associated with nausea and vomiting. Computerized tomography (CT) revealed a 10 × 7 × 6.5 cm left lower pole renal mass. Left radical nephrectomy was performed and revealed a tumor with uniform round to oval nuclei cells. The cells stained strongly positive for CD 99. The diagnosis was established as primary renal Ewing’s sarcoma. The patient refused the chemotherapy. Fortunately, he was free of the disease on regular clinical and radiological follow-up, the last follow up was 30 months post-nephrectomy.

**Conclusion:**

This case highlights the importance of proper surgical treatment and its role in the managing this type of malignancy especially in localized disease at presentation.

## Highlights

• Primary renal Ewing sarcoma is a rare tumor in adults.
• Proper surgical resection is the most important treatment step.
• Further studies needed to establish treatment for this tumor.

## 1 Introduction

Ewing’s sarcoma (EWS) is a rare form of a primitive, highly cellular malignant round-cell tumor of the bone and soft tissue described by Ewing [1]. EWS arise primarily from bones and they are rarely of extraskeletal origin [2]. It is most commonly found as a mass in the axial skeleton and in the soft tissue mass in the trunk [2].

Primary renal (EWS) is a rare tumor in adults. In 1975, Seemayer and colleagues were the first who described primary renal EWS and has since been sporadically documented in the literature [3,4]. The cellular origin of this type of cancer is unknown, however, it is thought to be derived from the neural and neural crest cells [5]. It is important to distinguish EWS from other entities that represent a renal mass because of its dismal prognosis as well as treatment implications. Only a few case reports and small case series of primary renal EWS were reported [4]. We present a case of primary renal EWS in 27-year-old male. He was treated surgically without evidence of disease after 30 months post nephrectomy.

This case study was performed and is being reported in line with the SCARE criteria [6].

## 2 Presentation of case

A 27-year-old Asian male; not known to have any medical illness; presented to our emergency department complaining of three days history of severe left flank pain associated with nausea and vomiting. No family history of malignancy was detected. Physical examination revealed left costovertebral angle tenderness. Laboratory investigations were conducted and were remarkable for elevated white blood cell count (13,000 ng/dl). Urine analysis and creatinine level were normal.

**Fig. 1:**
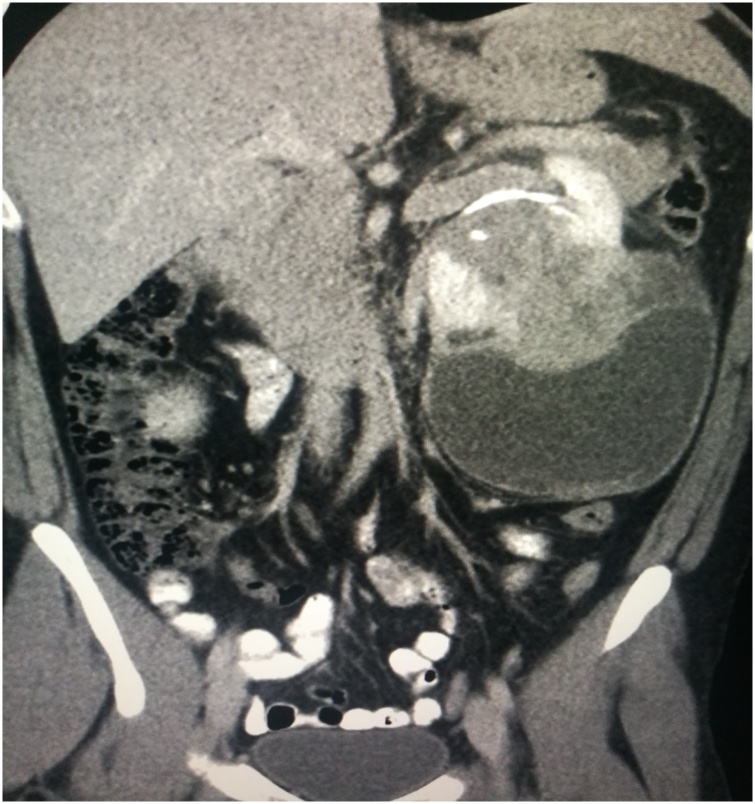
CT scan-Coronal view demonstrating huge left renal mass.

**Fig. 2:**
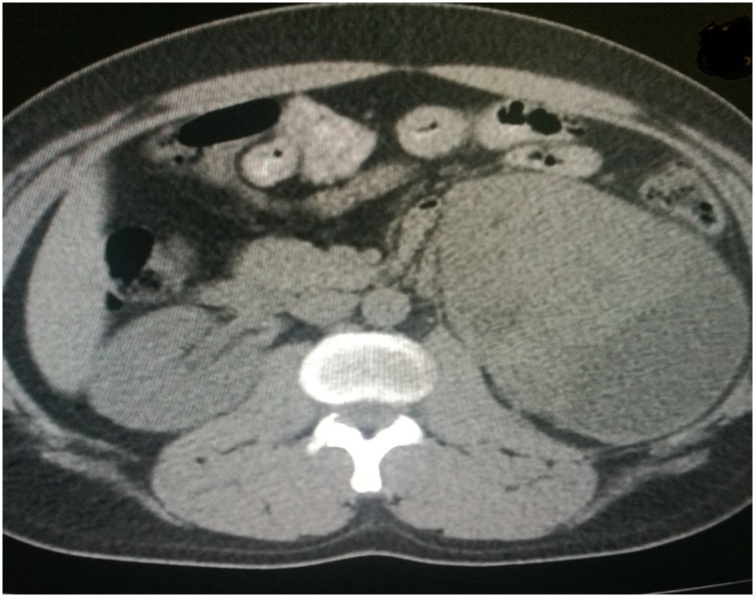
CT scan- axial view indicating left renal mass at the inferior pole.

An ultrasound was performed and revealed the presence of a large heterogenous cystic mass replacing the lower pole of the left kidney measured 12 × 10 cm. Computerized tomography (CT) revealed a 10 × 7 × 6.5 cm left lower pole renal mass. Part of the mass appeared in subcapsular location and causing significant mass effect over the left kidney with invasion into the renal pelvis and perirenal fascia (Figs. 1 and 2 ). The lungs and liver were clear without signs of metastasis.

He was admitted and a DMSA renal scan disclosed a split function of 24% for the left and 76% for the right. Bone scan revealed no scintigraphic evidence of osteoblastic or osteolytic bone metastasis. Then the decision was to take percutaneous renal biopsy, which revealed a small cell neoplastic tumor. Wilms tumor, pheochromocytoma, primary renal Ewing sarcoma and primary renal neuroblastoma were included as differential diagnosis.

**Fig. 3:**
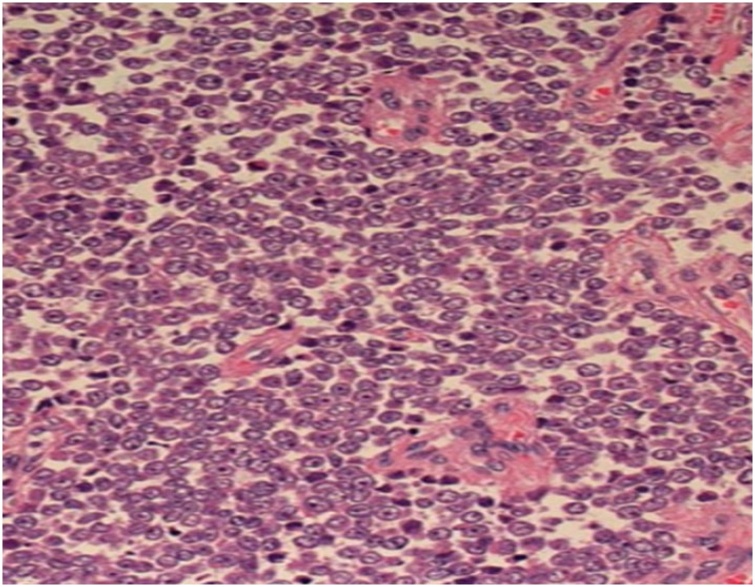
Microscopic view for the tumor showed uniform round small cells.

**Fig. 4:**
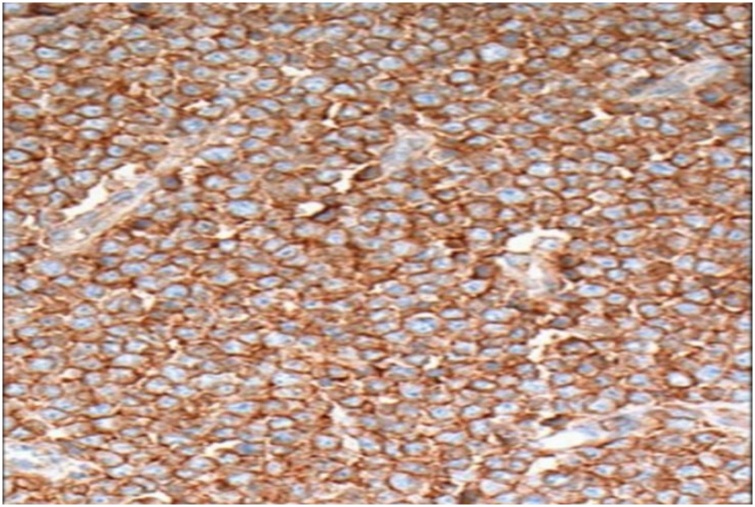
The tumor cells stained positively diffusely for CD99.

Later, left radical nephrectomy was performed. The renal mass weighed 572 g, measured 16 × 10 × 6 cm, and was surrounded by a thin membranous capsule (Unfortunately, the operational images were lost). On histopathological studies, the tumor was highly cellular; the tumor cells were uniform with round to oval nuclei, finely dispersed chromatin, inconspicuous nucleoli and ill-defined scant pink cytoplasm (Fig. 3). The cells stained strongly positive for CD 99 (Fig. 4). The diagnosis was established as renal EWS.

His post-operative course was uneventful other than some minor incisional pain. He was discharged on day 3 postoperative. He had no evidence of disease at 30 months after surgery and refused chemotherapy at that time.

## 3 Discussion

EWS of the kidney is an exceedingly rare entity. However, the true incidence may have been underestimated as the number of case reports is increasing in recent years, probably due to advanced immunohistochemistry [7]. Primary renal EWS was an aggressive malignant tumor and has male predominance with mean age of 28–34 years [[8], [9], [10]]. The clinical presentation is variable and includes flank pain (85%), palpable abdominal mass (60%), hematuria (37%) or weight loss (8%) [10,11]. Patients typically present at an advanced tumor stage [12]. The most common sites of metastasis are the lungs, followed by the liver and bone [12].

It is essential to distinguish renal EWS from other malignant neoplasms of kidney due to the prognostic point of view [12]. Small blue round cell tumor would be a diagnostic challenge in the field of renal histopathology because great number of other tumors have presented in a similar histopathological characteristic under microscope [[12], [13], [14]]. This group includes EWS or what is called primitive neuroectodermal tumors (PNET), non-Hodgkin Lymphoma, alveolar rhabdomyosarcoma, renal neuroblastoma, Wilms tumor, monophasic synovial sarcoma, desmoplastic small round cell tumor, carcinoid tumors, clear cell sarcoma of kidney [5,[12], [13], [14]]. The immunohistochemical pattern of renal EWS is similar to that seen in other locations [15,16]. Proteins encoded by the MIC2 gene, most commonly CD99 or O-13, are the most commonly expressed markers [15,16]. CD99 immunohistochemistry is positive in more than 90% of ES/PNET [15,16].

Imaging findings in EWS include a large, ill-defined renal mass, often with heterogeneous contrast enhancement with areas of necrosis and hemorrhage [[8], [9], [10], [11], [12]]. The imaging features are in general nonspecific and the diagnosis, although rare, should be entertained whenever a young patient presents with a large renal mass.

Because of the rarity of this tumor, there is no standardized treatment strategy. The primary modality is surgical excision [8,9]. The 2-year overall survival of patients who undergo surgery is 80%, compared with 30% for those who do not [8]. Approximately half of patients receive neoadjuvant or adjuvant chemotherapy. Because of biologic similarities to EWS at other sites, the cases primary to the kidney are treated in a similar fashion [9]. The five common chemotherapeutic agents are doxorubicin, vincristine, cyclophosphamide, ifosfamide, and etoposide [17]. Rowe et al reported in his review that only three cases did not report the administration of chemotherapy [4,[18], [19], [20]]. Two of them reported a relapse while the third was alive without the disease 12 months after the nephrectomy [[18], [19], [20]]. In the presented case, we consulted and advice the patient to have chemotherapy, but he refused. Fortunately, he was free of the disease on regular clinical and radiological follow-up, the last follow up was 30 months post-nephrectomy. To our knowledge, this article reported the successful surgical treatment for EWS without adjuvant chemotherapy with the longest period of resolution without any relapse.

## 4 Conclusion

We report yet another case of primary renal EWS, in hope of expanding the knowledge of a rare occurrence and increasing the demand for further research about the etiology, clinical manifestation, prognostic factors and treatment modalities. This case also highlights the importance of proper surgical treatment and its role in the managing this type of malignancy especially in localized disease at presentation.

## Funding

No funding.

## Ethical approval

The institutional review board is not required.

## Consent

The patient gave his consent for publication of this Case report and any accompanying images. None of the images contains any patient`s identifiers.

## Author’s contribution

All authors contributed significantly and in agreement with the content of the article. Abdalla, Tashtush, and Aleshawi collected all data and photographs to draft the manuscript. Talahmeh was the urologist who perform the surgery. Abdalla and Alawneh were the radiologist. Tashtush, Abdalla, and Aleshawi wrote the manuscript for submission. All authors presented substantial contributions to the article and participated of correction and final approval of the version to be submitted.

## Registration of research studies

None.

## Guarantor

Dr Khalid Abdalla.

## Provenance and peer review

Not commissioned, externally peer-reviewed.

## Declaration of Competing Interest

The authors declare that they have no competing interests.
